# Experimental investigation into the effects of composition and microstructure on the tensile properties and failure characteristics of different gypsum rocks

**DOI:** 10.1038/s41598-021-93947-6

**Published:** 2021-07-15

**Authors:** Hongfa Ma, Shaojie Chen, Yanqi Song, Dawei Yin, Xiangshang Li, Xiaolong Li

**Affiliations:** 1 School of Mechanics and Civil Engineering, China University of Mining and Technology (Beijing), Beijing, 100083 China; 2 State Key Laboratory of Mine Disaster Prevention and Control, Shandong University of Science and Technology, Qingdao, 266590 China; 3 State Key Laboratory for GeoMechanics and Deep Underground Engineering, China University of Mining and Technology (Beijing), Beijing, 100083 China

**Keywords:** Civil engineering, Mechanical engineering

## Abstract

The present work investigated the differences in the composition and internal microstructure of four types gypsum rock—fiber gypsum, transparent gypsum, alabaster, and ordinary gypsum by X-ray fluorescence spectrometry, X-ray diffraction, scanning electron microscope and Brazilian split test, and analyzed its effects on the tensile strength and fracture characteristics of gypsum rock. For alabaster, fiber gypsum, transparent gypsum, and ordinary gypsum, CaSO_4_·2H_2_O is the main component with 72.78%, 72.72%, 72.57%, and 71.51% content, and tensile strength of 1.79, 2.22, 3.22, and 4.35 MPa, respectively. In addition, the fracture line is arc-shaped, vertical, and zigzag for fiber gypsum, ordinary and transparent gypsums, and alabaster, respectively. On the microscopic level, fiber gypsum has an evident striated structure while the gradual increased pore development for alabaster, transparent gypsum, and ordinary gypsum. Gypsum rock has an obvious layered crystal structure with the increase of CaSO_4_·2H_2_O, contributing to the phenomenon with a larger grain size and lower tensile strength. In addition, the number of particles for alabaster, transparent gypsum, and ordinary gypsum increased in turn, while their particle size decreased uniformly, indicating that the lower CaSO_4_·2H_2_O content, the more sufficient energy accumulation and release. This paper can provide a theoretical basis for the analysis of the mechanical properties of rocks with different mineral composition and contribute to the design for different ore grades mining.

## Introduction

Gypsum has long been considered an important raw material for engineering construction. Based on its microstructure and component, gypsum rocks can be classified into several kinds. For the mining of underground gypsum, room and pillar mining method is commonly adopted to obtain by “retaining pillars and mining room” to treat goaf^1–6^. After ore extraction, original rock stress redistributes, thereby the mechanical properties of the retained pillars are closely related to the stability of the goaf. Existing research shows that the tensile strength of a rock is significantly lower than its compressive strength. Thus, the tensile performance of a rock is a critical factor in evaluating its stability in an engineering system^7,8^. In addition, the mineral composition and microstructure characteristics of a rock directly affect its mechanical properties^9–12^, thereby making them key factors in rock damage and fracture.

There are direct and indirect methods often used in measuring tensile strength. To avoid the irregularity of tensile stress, indirect tensile method or Brazilian disc split test is often used locally and abroad for rock materials^13–18^. This method investigates the influence of different loading rates on the tensile strength of a rock in a micro view and energy evolution^19–21^. Rafiei Renani and Martin^22^ studied the effect of rock size on its tensile strength by strength statistical theory and fracture mechanics. Masoumi et al.^23^ found that all rock types follow a generalized size effect concept, in which the strength decreases with the increased size, through indirect tensile tests from different geological origins. Ofoegbu et al.^24^ studied the law of crack initiation and propagation in rock splitting tests by numerical simulation and discussed the fracture mechanism of the local failure area.

The mechanical behavior of a rock is significantly influenced by its mineral composition and microstructure. He et al.^25^ analyzed the influence of clay, cement, and mineral composition on the strength and deformation of sandstone. Wanniarachchi et al.^26^ studied the effect of mineral composition on the mechanical properties of shale gas reservoir, showing that tensile strength increased with kaolinite and calcite contents. Martin et al.^27^ examined the relationship between the rock composition and natural fracture strength, thereby noting that the natural fracture strength is directly proportional to calcium, silicon/aluminum, and total organic carbon content, and indirectly proportional to silicon and aluminum. Zhang et al.^28^ noted the significant effect of the clay mineral composition in the mechanical parameters of coal and rock. Meng et al.^29,30^, Zuo et al.^31^ and Wang et al.^32^ studied the interaction between the composition and macroscopic mechanical properties of rocks, showing its direct relationship to its microstructure. Duncan^33^, Chen et al.^34^, Gao et al.^35^, and Du et al.^36^ investigated the influence of the mineral particle size on the mechanical behavior of sylvite rich rock, sandstone, and concrete, respectively. Přikryl^37^, Lindqvist et al.^38^, and Johansson^39^ examined the influence of the particle morphology, cementation degree, and microcrack distribution on the mechanical properties of rocks, respectively.

There is a large number of existing literatures on the influence of composition and microstructure individually on the mechanical behavior of rocks. Although these are highly significant, there are only few comprehensive studies that consider both factors. Moreover, there are limited reports on the tensile strength of rocks, which is an important parameter for the characterization of its mechanical properties. Thus, in this study, the composition and internal microstructure of four kinds of gypsum rocks, namely fiber gypsum, transparent gypsum, alabaster, and ordinary gypsum, were analyzed. Brazilian split experiments were carried out to obtain their physical parameters and tensile strength. The correlation between the composition and microstructure characteristics of gypsum rocks and their tensile strength were discussed.

## Materials and methods

### Experimental process

**Figure 1:**
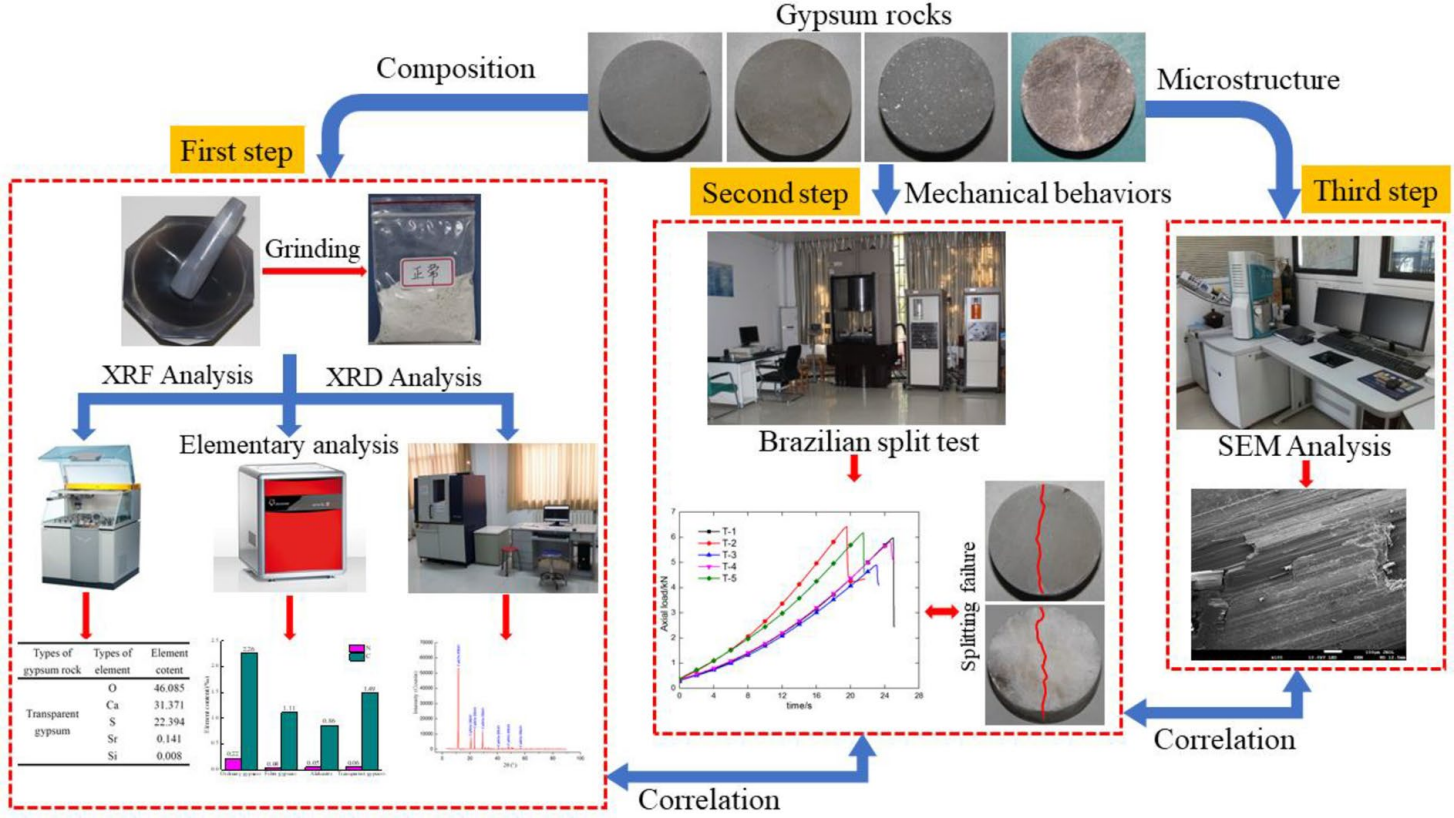
Experimental scheme of the tests for the characterization and the mechanical properties of gypsum rocks: X-ray fluorescence spectrometry (XRF), X-ray diffraction (XRD), Elemental analyzer, scanning electron microscopy (SEM), and Brazilian split test for the mineral composition, microstructure, and tensile strength of gypsum rocks, respectively. (The figure is just a screenshot which contains all the equipment and test pictures used in this paper, and is not generated by other software.)

To study the effects of microstructure and composition on the tensile strength of gypsum rocks, X-ray fluorescence spectrometry (XRF), elementary analysis and X-ray diffraction (XRD) were used to analyze the mineral composition. Brazilian split test was also carried out to obtain the tensile strength of gypsum rock by MTS815 universal testing machine. Scanning electron microscopy (SEM) was used to investigate the failure microstructure of gypsum rocks. The scheme of the experimental process is shown in Fig. 1. The details of the samples and equipment are introduced in the next section.

### Materials and sampling

Gypsum rock samples were obtained from Luneng Taishan gypsum mine, Shangdong Province, China, for alabaster, transparent gypsum, and ordinary gypsum, and from Jingmen gypsum mine, Hubei Province, China, for fiber gypsum. To ensure that the test results replicate actual underground conditions, the rocks were packed with a fresh-keeping film after removal and during its transport to the laboratory.

#### XRF and XRD samples

XRF, XRD and elementary analysis test were carried out to explore the difference of the mineral compositions of different gypsum rocks. Before the experiment, representative broken rock blocks were removed for the four kinds of gypsum rocks, respectively. The representative samples were crushed and ground into powder solid with a particle size of approximately 48 µm. A total of 10 g was collected and sealed for each kind of gypsum rock sample.

#### Brazilian disc samples

**Figure 2:**
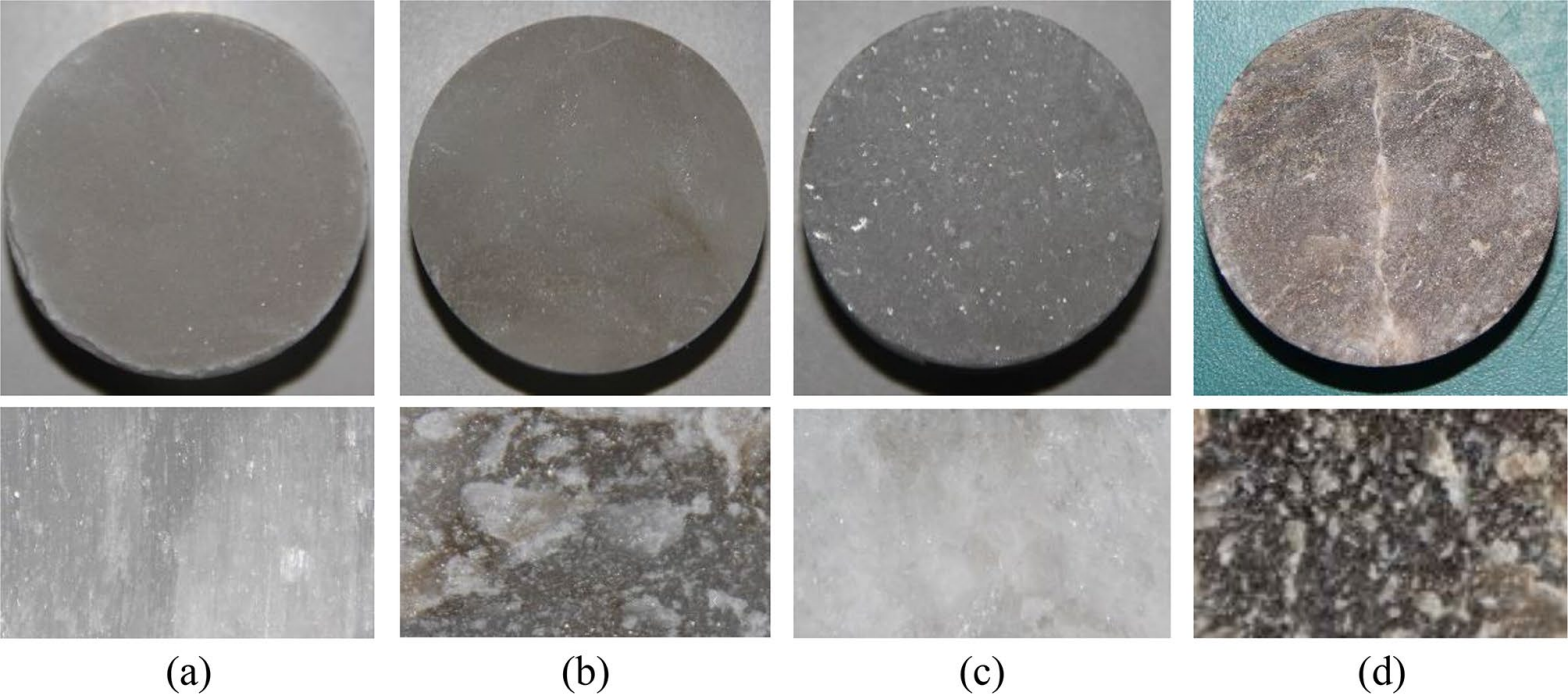
Surface and interior characteristics of the four types of gypsum rock samples: (**a**) fiber gypsum with transparent features and longitudinal fibrous structures, (**b**) transparent gypsum with local dark features and white flaky structures, (**c**) alabaster with white transparent features and transparent granules, (**d**) ordinary gypsum with whole dark features and local white flaky structures. (The Figure contains the pictures of four kinds of gypsum rock obtained by camera, and is not generated by other software.)

The Brazilian split test samples were obtained as follows. The four types gypsum rocks were obtained on site and processed into samples sized Φ 50 × 25 mm by coring, cutting, grinding, and other processes. To reduce the deviations of the test results, samples with obvious defects were removed after macroscopic analysis. Sonic velocity measurement analysis was conducted and the rock samples with similar wave velocities were selected as the test samples for each group. The longitudinal section characteristics of the gypsum rock samples are shown in Fig. 2.

As shown in Fig. 2, the experimental samples include three kinds of high-purity gypsum rocks—fiber gypsum, transparent gypsum, and alabaster, low-ore grade transparent gypsum rock, referred to as ordinary gypsum, was selected for the comparative analysis. In addition, fiber gypsum has a translucent characteristic with compact striped fiber structure distributed vertically along the axial direction. Transparent gypsum has a dense and translucent appearance with dark color on some areas, and with obvious crystalline particles. For alabaster, there are large white crystal particles distributed with a low compactness visible to the naked eye, thereby presenting its white color. Ordinary gypsum has high density and dark gray appearance with white spots and a clastic feature similar to that of transparent gypsum.

#### SEM samples

Four types of gypsum rock blocks were crushed to obtain rectangular test samples with a side length less than 1 cm and one untreated main plane (length × width), and each group had at least two finished rock samples. The main plane was considered as the observation surface, meanwhile, the debris and surface residue attached to the surface was removed by a suction balloon and in high-purity alcohol immersion, respectively. Finally, gold powder was sprayed on the sample to increase the conductivity.

#### Material composition analysis equipment

XRD tests were performed by the D/max 2500 PC XRD device (radiation, 2 h = 3°–90°) with an operating voltage of 40 kV, emission current of 40 mA, and step size of 0.02. The XRF element detection equipment is PANalytical Axios, Range of measurable elements: 11 Na-92 U. The elementary analysis test equipment is Elementar vario PYRO which can be used to analyze the isotopic ratio of O, H and C, N, S.

#### SEM equipment

JSM-6510LV high- and low-vacuum SEM was used to analyze the microstructure of the gypsum rocks after fracture. Its high-vacuum resolution is 3 nm (30 kV), 8 nm (3 kV), 15 nm (1 kV), respectively, and its low-vacuum resolution is 4.0 nm (30 kV).

#### Mechanical test equipment

Brazilian split test is the most commonly used method to obtain the tensile strength of rock. In this study, MTS815 universal testing machine was used. It is driven by hydraulic control with 1500 kN maximum load, up to ± 0.1% precision, and can perform various tests for mechanical properties. The loading mode of the samples was controlled by displacement with the loading rate set to 0.01 mm/s.

#### Strength calculation principle

In the Brazilian split test, especially for sandstone and other transversely isotropic rocks, there are two methods in calculating the indirect tensile strength^40–42^, and among which the more commonly used is shown in Eqs. (1)

1 $$\sigma _{t} {\text{ = }}\frac{{2P}}{{\pi Dt}}$$

where *σ*_*t*_ is the tensile strength of the rock, *P* is the failure load, *D* is the diameter of the Brazilian disc sample, *t* is the height of the Brazilian disc sample.

Equation (1) is commonly used because of its simplicity. However, the test materials should meet two criteria: (1) homogeneity and isotropy, and (2) macro failure crack at the center of the disk. In this study, only the striped structure of fiber gypsum rocks was characterized by bedding. In addition, this test aims to compare the different laws on the tensile strength of the four kinds gypsum rocks. Therefore, Eq. (1) could satisfy the research needs.

## Results and analysis

### Mineral composition

XRF element analysis and XRD were carried out to investigate the differences in the composition of different gypsum rocks and the influence of composition on the tensile strength.

#### XRF element analysis

XRF was performed on each group of samples to obtain the elemental composition and content of the gypsum rocks. In addition, it was determined that the composition of the rock included components in the form of oxides, and the element contents are shown in Table 1.

**Table 1:** XRF results.

Gypsum rock	Mass fraction of elements and corresponding oxides										
	O	Ca	S	Si	Al	Sr	Fe	Mg	K	P	Cr
Ordinary gypsum	45.99	31.24	22.00	0.28	0.15	0.14	0.09	0.09	0.02	0.01	–
	–	CaO	SO_3_	SiO_2_	Al_2_O_3_	SrO	MgO	Fe_2_O_3_	K_2_O	P_2_O_5_	–
	–	43.71	54.95	0.60	0.28	0.17	0.15	0.13	0.02	0.01	–
Alabaster	46.09	31.37	22.39	0.01	–	0.14	–	–	–	–	–
	–	CaO	SO_3_	SiO_2_	–	SrO	–	–	–	–	–
	–	43.89	55.92	0.02	–	0.17	–	–	–	–	–
Fiber gypsum	46.09	31.50	22.38	–	–	0.02	–	–	–	–	0.02
	–	CaO	SO_3_	–	–	SrO	–	–	–	–	Cr_2_O_3_
	–	44.08	55.88	–	–	0.02	–	–	–	–	0.02
Transparent gypsum	46.04	31.44	22.33	0.01	–	0.17	–	–	–	0.01	
	–	CaO	SO_3_	SiO_2_	–	SrO	–	–	–	P_2_O_5_	Cr_2_O_3_
	–	43.99	55.76	0.02	–	0.20	–	–	–	0.02	0.02

Table 1 shows that the number of elements in ordinary gypsum rock is higher than that in the other three gypsum rocks; however, the four types of gypsum rock comprise the same main elements, which include, in decreasing order, O, Ca and S. The contents of O, Ca, and S in ordinary gypsum are 45.99%, 31.24%, and 22.00%, respectively, while there is little Fe and P in ordinary gypsum rock, which may contribute to the dark characteristics of the rock. SiO_2_ is present in ordinary gypsum, transparent gypsum and alabaster (in order of decreasing content), but it is not found in fiber gypsum because of the different places of origin. Moreover, because the content of S, which has a relative atomic mass of 64, is lower in all gypsum rocks than that of Ca, which has a relative atomic mass of 40, so there may be other composition that contains Ca in the rock. The four kinds of gypsum rocks contain little SrO, which means that all the gypsum rocks may have a small amount of calcite (usually containing Sr).

#### XRD phase identification

**Figure 3:**
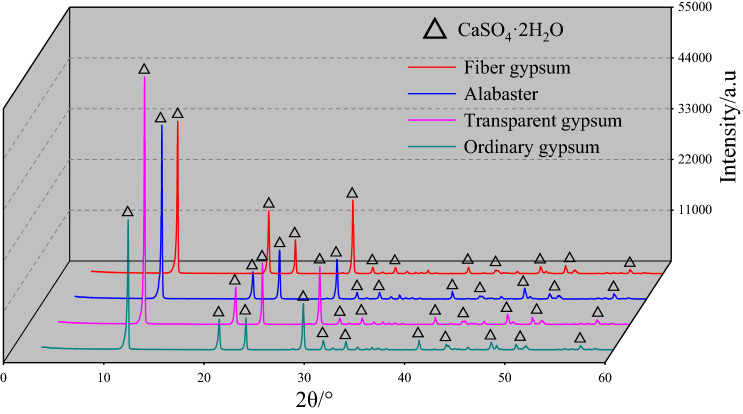
X-ray diffraction curves of the four kinds of gypsum rocks. (The Figure is just obtained from the process of XRD test data by the data mapping software of Origin 2018, https://www.originlab.com/index.aspx.)

To explore the differences in material composition of the four types of gypsum rocks, material analysis was carried out through XRD. The characteristic patterns are shown in Fig. 3.

**Figure 4:**
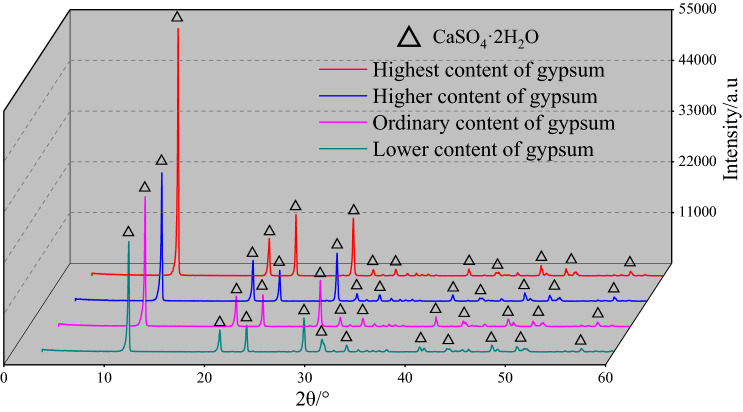
XRD curves of different grade of transparent gypsum rock. (The Figure is just obtained from the process of XRD test data by the data mapping software of Origin 2018, https://www.originlab.com/index.aspx, and the generation process of the figure is consistent with Figs. 5 and 6.)

The XRD curves of the four types of gypsum rocks are relatively similar with strong first four diffraction peaks and minimal difference in the diffraction angles of the main peaks. The detection results showed CaSO_4_·2H_2_O is the main component of gypsum rocks. The main difference for the curves is the intensity of the first diffraction peaks, which indicates variation in the crystalline-preferred crystal orientation^43^. The peak intensity of the first diffraction peak is higher for transparent gypsum than for ordinary gypsum. Therefore, to explore the impact of the ore grade of the gypsum rock on the intensity of the first diffraction peak, XRD experiments were also performed on different grades of transparent gypsum rock, as shown in Fig. 4, and the statistical results for different gypsum rocks processed by MDI Jade 6.0 are shown in Table 2.

**Table 2:** Data retrieval results of MDI Jade 6.0 software.

Gypsum rock type	Phase name	Molecular formula	Θ^a^/°	D^b^/nm	Crystal size/nm
Fiber gypsum	Gypsum. syn	CaSO_4_·2H_2_O	11.607	7.6236	929
Transparent gypsum			11.610	7.6218	896
Alabaster			11.690	7.5641	990
Ordinary gypsum			11.587	7.6309	691

^a^θ is the diffraction angle of the main peak; ^b^D is the crystal surface spacing.

As shown in Fig. 4, the XRD peak curves are similar for the four different grades of transparent gypsum rocks. CaSO_4_·2H_2_O is noted as the main component. The peak intensity of the first diffraction peaks increased with increasing CaSO_4_·2H_2_O content, while the other three main diffraction peaks exhibited similar changes. Thus, it can be concluded that the CaSO_4_·2H_2_O content affects the diffraction intensity in the XRD curves.

Table 2 shows the results from the MDI Jade 6.0 software which was used to process and analyze the experimental data. Based on the XRF element analysis results, gypsum. syn which has a molecular formula of CaSO_4_·2H_2_O, was found to be the main component in the four types of gypsum rocks. In addition, the gypsum rocks had a stable crystal face spacing of approximately 7.60 nm with obvious differences in the crystal sizes for each type. The crystal size was at the micron level, which is significantly larger than the crystal face spacing.

**Figure 5:**
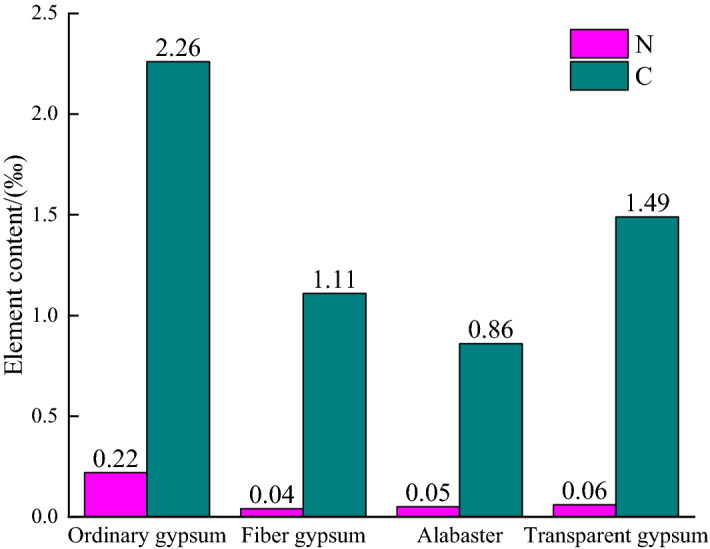
The content of C and N elements in different gypsum rocks based on element analysis.

Because XRF cannot measure the content of elements smaller than Na, the XRD result shows that all the gypsum rocks have a main composition of CaSO_4_·2H_2_O; however, the presence of carbonates, such as CaCO_3_, cannot be ruled out. Thus, to finally obtain the content of CaSO_4_·2H_2_O for each kind of gypsum rock, elemental analysis was carried out by an element analyzer to obtain the content of C, and the test results are shown Fig. 5.

Figure 5 shows that the C contents in the four gypsum samples were less than 3‰, which indicates that there may be few carbonates in any of the gypsum rocks and that they do not have a significant impact on the composition of the rock. It is worth noting that the C contents in ordinary gypsum, transparent gypsum, fiber gypsum and alabaster decrease in turn, which is the same trend as was observed for the S content of the rocks. In addition, the contents of N in the rock were all less than 0.25‰, which should not be analyzed in the later discussion of gypsum rock compositions.

#### Calculation of CaSO_4_·2H_2_O content

According to the XRF, XRD and elementary analysis experiments, CaSO_4_·2H_2_O was the major component of the four kinds of gypsum rocks. However, the relationship between the elements and composition of other substances was not completely determined. Because we determined the proportions of the elements (e.g., Ca:S:O ratio of 1:1:6 in CaSO_4_·2H_2_O) and obtained the content of S, we can calculate the CaSO_4_·2H_2_O content by the following equation:

2 $$\begin{gathered} {\text{ Chemical relation: }}S \leftrightarrow CaSO_{4} \cdot 2H_{2} O \hfill \\ {\text{Relative molecular mass: 64 208}} \hfill \\ {\text{ Mass fraction: ? ?}} \hfill \\ \end{gathered}$$

Through elemental analysis, we obtained the result that there is little C in each type of gypsum rock. Based on the XRF results, we obtained the content of S for each gypsum rock; therefore, we can conclude that the CaSO_4_·2H_2_O content by Eq. (2) of the four gypsum rocks is as follows: fiber gypsum (72.72%), transparent gypsum (72.57%), alabaster (72.78%), and ordinary gypsum (71.51%). The results show that there is little difference in the contents of CaSO_4_·2H_2_O in the four kinds of gypsum rock and that the dark characteristics of ordinary gypsum and transparent gypsum rock may be caused by the low contents of other elements.

### Tensile test results

**Figure 6:**
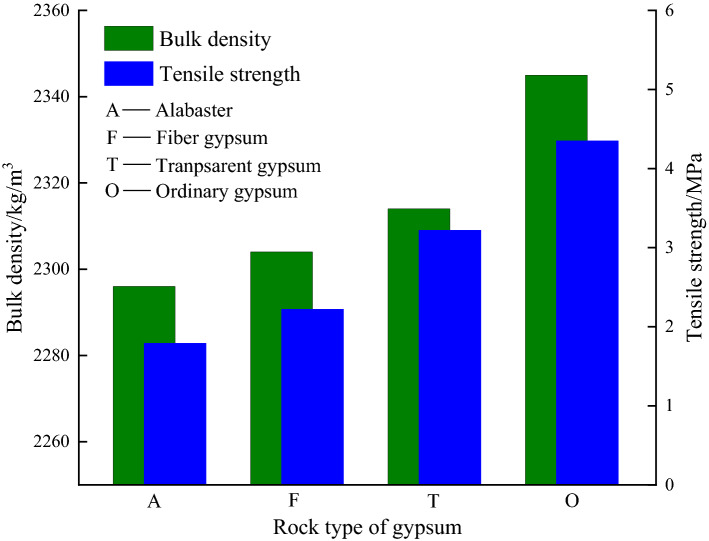
Tensile strength and density of the four types of gypsum rocks: alabaster, fiber gypsum, transparent gypsum, and ordinary gypsum.

The results of the Brazilian split test of the four kinds gypsum rocks are shown in Table 3 and the difference of tensile strength and density are shown in Fig. 6.

**Table 3:** Brazilian splitting test results.

Gypsum type	Number	Diameter/mm	Height/mm	Mass/g	Bulk density/kg/m^3^	Tensile strength/MPa
Transparent gypsum	T-1	49.04	22.81	99.87	2319	3.41
	T-2	49.04	25.32	110.16	2305	3.30
	T-3	48.80	23.67	102.72	2321	2.70
	T-4	48.92	23.22	100.63	2307	3.27
	T-5	49.04	23.62	103.32	2317	3.40
	Mean				2314	3.22
Fiber gypsum	W-1	48.84	25.74	111.12	2305	1.64
	W-2	48.90	25.34	109.49	2302	2.37
	W-3	48.84	24.85	107.37	2307	1.62
	W-4	48.84	24.76	107.02	2308	2.88
	W-5	48.86	25.29	109.03	2300	2.61
	Mean				2304	2.22
Alabaster	X-1	48.78	24.48	101.11	2277	1.65
	X-2	48.74	25.28	108.36	2298	1.30
	X-3	48.64	22.91	98.57	2316	2.10
	X-4	48.84	23.17	99.78	2299	2.44
	X-5	49.00	23.01	99.36	2291	1.46
	Mean				2296	1.79
Ordinary gypsum	B-1	48.92	24.63	107	2312	4.61
	B-2	49.02	23.06	103	2367	4.08
	B-3	49.10	23.35	105	2376	4.40
	B-4	49.14	24.95	110	2325	4.31
	Mean				2345	4.35

Figure 6 shows that the relatively large tensile strength of transparent gypsum can be attributed to its relatively compact internal structure and small grain size. Meanwhile, the striped surface with sliver and layered structures in the radial direction of the fiber gypsum are easily separated under tensile stress, leading to its lower tensile strength. The large internal particles in alabaster contributed to the stress concentration under uniaxial loading, causing premature failure of the rock sample. In comparison to transparent gypsum, ordinary gypsum has a similar structure, better compactness, and internal cementation, leading to a slight improvement in the overall tensile strength. These structure differences were further studied by SEM.

From Table 3 and Fig. 6, the density variations of the different gypsum rocks are similar to their tensile strength. The density of ordinary gypsum, transparent gypsum, fiber gypsum, and alabaster declines in turn. With a similar rock composition, the changes in density reflect the difference in the internal structure and compactness of each gypsum rocks.

### Failure characteristics of gypsum rock samples

#### Macroscopic failure characteristics

**Figure 7:**
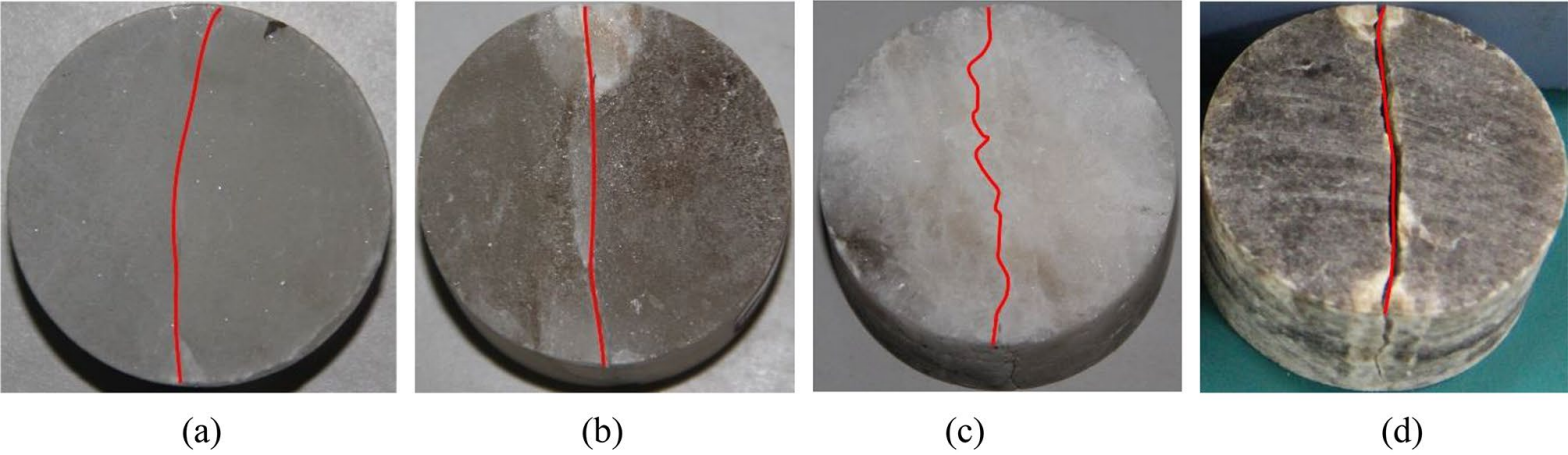
Macro splitting failure characteristics of the four types of gypsum rocks: (**a**) fiber gypsum, (**b**) transparent gypsum, (**c**) alabaster, and (**d**) ordinary gypsum. The red lines represent the fracture lines. (The figure which is not generated by other software are just the photographs of four kinds gypsum rock samples obtained by the camera, and the red line in each sample is drawn to present the difference in failure characteristics for different gypsum rock.)

The indirect tensile failure characteristics with the fracture lines of the four types gypsum rock are shown in Fig. 7. The fracture line of fiber gypsum is relatively smooth traversing through the entire specimen with an arc shape, indicating the extension of the fracture line along its fibrous arcuate structure. Transparent and ordinary gypsums have a similar fracture line that distributed along the center of the specimen without evident inflections. The fracture line of alabaster is zigzag, extending along the edge of large crystal particles, and traversing through the crystal particles in some areas.

#### Microscopic failure characteristics

**Figure 8:**
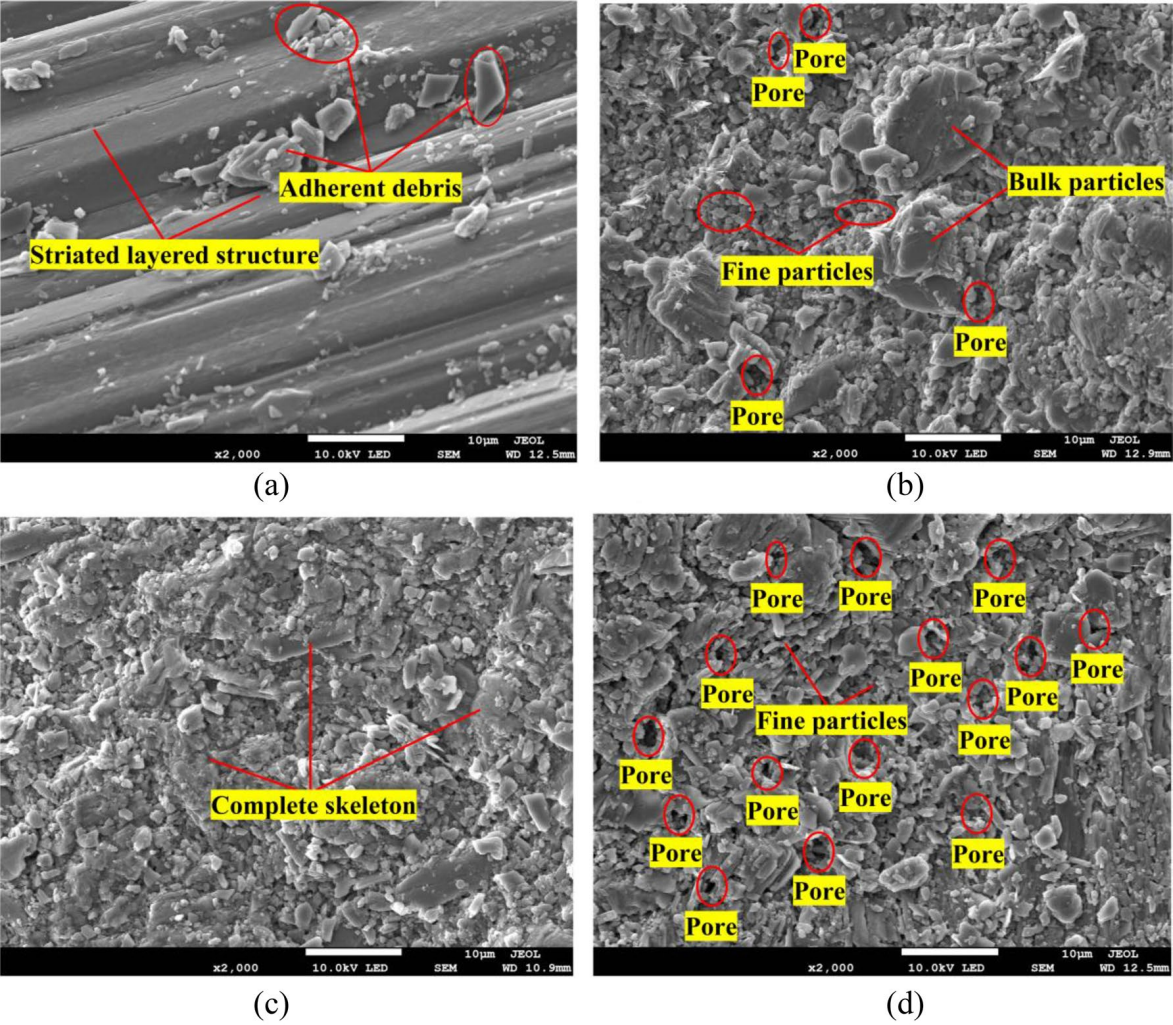
Microscopic failure characteristics at a magnification of 2000 for the four types of gypsum rocks investigated: (**a**) fiber gypsum, (**b**) transparent gypsum, (**c**) alabaster, and (**d**) ordinary gypsum. (The figure which is not generated by other software is just the picture obtained by the SEM test, meanwhile, the red lines and black words on yellow background are added by the Microsoft office 2016, https://www.microsoft.com/zh-cn/microsoft-365/previous-versions/microsoft-office-2016.)

Figure 8 shows the micromorphological characteristics of the four types of gypsum rocks at a magnification of 2000. Even with the striped structure of fiber gypsum, the connections between fringes are relatively compact with several clastic particles on the surface. Large particles can be observed in transparent gypsum but micro clastic particles still dominated the structure; moreover, the degree of cementation between the particles is relatively low with notable pores in some areas. Alabaster has a sturdy surface with undamaged veined skeleton and without pores, indicating characterization by local failure. The particle size of ordinary gypsum is relatively small with good homogeneity and developed pores.

## Discussions

### Correlation analysis of gypsum rock components and tensile strength

**Figure 9:**
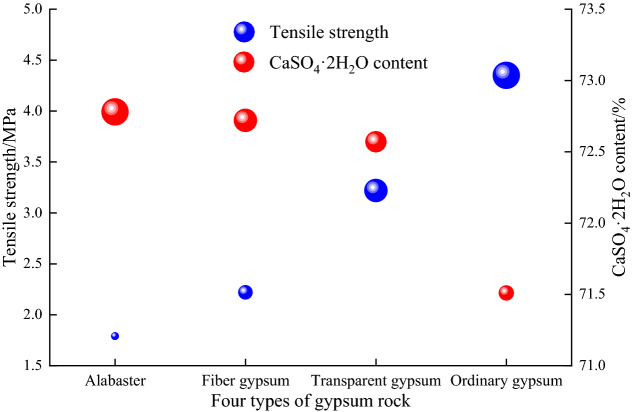
The relationship between CaSO_4_·2H_2_O content and tensile strength of gypsum rocks. The size of the ball is consistent with the value of tensile strength and the content of CaSO_4_·2H_2_O.

According to the analysis of the material composition and Brazilian split test, there may be a relationship between the CaSO_4_·2H_2_O content and tensile strength of the gypsum rocks. Particularly, the higher the CaSO_4_·2H_2_O content is, the lower the tensile strength. Thus, the relationship between the CaSO_4_·2H_2_O content and tensile strength of different gypsum rocks was determined by the test results, as shown in Fig. 9.

Figure 9 shows that the higher the CaSO_4_·2H_2_O content is, the lower the tensile strength. However, for ordinary gypsum, transparent gypsum, fiber gypsum and alabaster, the CaSO_4_·2H_2_O content increases gradually with contents of 71.51%, 72.57%, 72.72%, 72.78%, respectively, but the tensile strengths of the four types gypsum samples are 4.35 MPa, 3.22 MPa, 2.22 MPa, 1.79 MPa, respectively. In addition, the CaSO_4_·2H_2_O content decreases from the highest 72.78% to the lowest 71.51%, which represents a 1.74% decrease, though the decrease in tensile strength is 58.85%. The results show that the influence of the CaSO_4_·2H_2_O content on the tensile strength of gypsum may be relatively small and that the microstructural differences caused by the CaSO_4_·2H_2_O content may account for the differences in the tensile strength of different gypsum rocks.

### Correlation analysis of the tensile strength and microstructure

#### Molecular structure and grain characteristics

**Figure 10:**
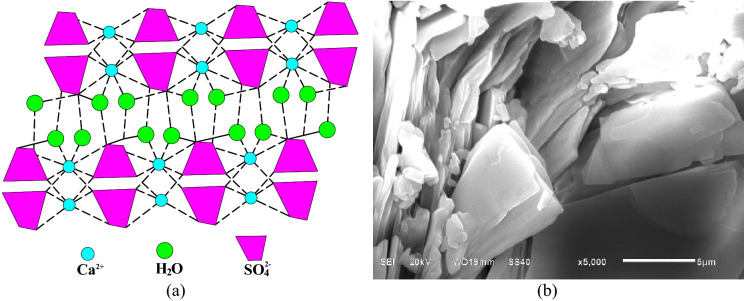
(**a**) Molecular space arrangement and (**b**) microstructure with layered structure at the magnification of 5000 of gypsum rock. (This figure (a) is a picture drawn by the Microsoft office 2016, https://www.microsoft.com/zh-cn/microsoft-365/previous-versions/microsoft-office-2016, the figure(b)is not generated by other software but obtained by the SEM test.)

Rock is a complex mineral aggregate with its own particular internal material composition and spatial structure^44^. From the diagenetic conditions and grain size of the microstructure, the main material composition of the gypsum rocks was found to be CaSO_4_·2H_2_O. The effect of the microstructure on the tensile strength of the gypsum rocks and its mechanisms were analyzed based on the experimental results. Figure 10 shows the CaSO_4_·2H_2_O molecular space arrangement and the microstructure of gypsum rock.

From Fig. 10a that the CaSO_4_·2H_2_O molecules are arranged in layers with crystal water connected and distributed in the middle by weak chemical bonds^45^. The layered structure formed in the gypsum is shown in Fig. 10b. When load is applied, a tensile stress perpendicular to the layer direction is generated, leading to tensile failure. With a higher CaSO_4_·2H_2_O content, there is a more evident concentrate stress phenomenon, which makes the rock sample more prone to damage and decreases the tensile strength on a macro level.

Gypsum rock has a complete crystal structure with different grain size for each type gypsum rock. Many scholars noted the inverse correlation between the strength of nonporous rocks and the size of crystal particles^46–49^. From Table 2, the grain sizes of ordinary gypsum, transparent gypsum, fiber gypsum, and alabaster are 691, 896, 929, and 990 nm, respectively. In addition, the tensile strength of gypsum is inversely proportional to the crystal size except for fiber gypsum due to its striated structure.

#### Particle granularity of fractured rock samples

**Figure 11:**
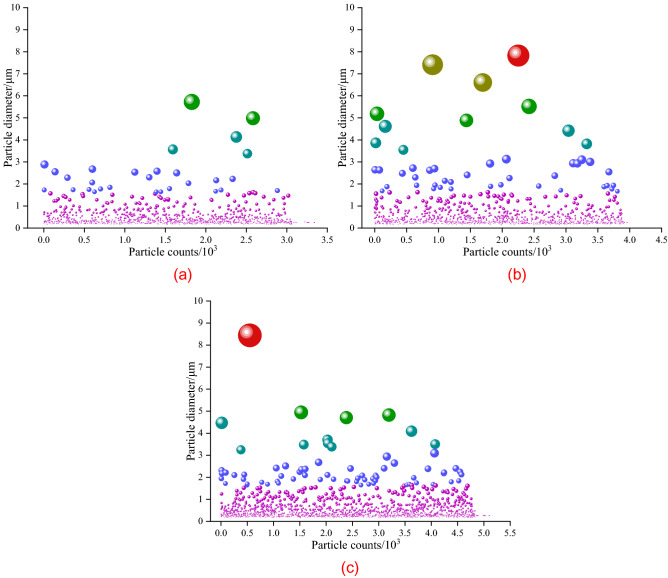
Statistics of the fracture particles of gypsum rocks based on the scanning electron microscopy images: (**a**) alabaster, (**b**) transparent gypsum, and (**c**) ordinary gypsum. The size of the ball is consistent with the diameter of the fracture particle.

To further explore the particle characteristics after failure of the four kinds of gypsum rocks, the SEM images were analyzed by Image Pro Plus and the diameters of the failure particles were measured and counted. The results are shown in Fig. 11. The fracture of fiber gypsum rock was along its fiber interlayer without obvious particle characteristics and only some clastic particles adhered to its surface. Thus, the particle size statistics was only applicable for the other three kind gypsum rocks.

It can be seen from Fig. 11 that the three gypsum rocks have different particle size distribution characteristics. Under the same statistical area, the particle diameter of alabaster is below 2 mm with few particles in the range of 4–8 mm. From the SEM image in Fig. 8b, there are large particles in transparent gypsum rock, differing from the statistical analysis, in which the main fractured particle diameter range is less than 2 mm. The particle size of ordinary gypsum rock is relatively uniform with a main distribution range below 2 mm and all particle diameters are less than 6 mm.

Under the same statistical area, the total number of statistical particles can reflect the fracture degree of gypsum rock. The number of statistical particles of alabaster, transparent gypsum, and ordinary gypsum are approximately 3000, 4000, and 5000, respectively. The result indicates the greater fracture degree and more thorough energy release for ordinary gypsum rock under equal loading condition. Compared with the SEM images, alabaster has significantly less particles in micro failure, further verifying the minimal change of the overall structure of alabaster during fracture. In addition, there is a linear relationship between the tensile strength of the group samples and the statistical number of particles after failure within the same statistical range, which appears the greater the tensile strength, the more the number of fracture particles.

#### Microstructural characteristics of fractured rock samples

According to the analysis in Figs. 7 and 8, there are large differences in the microstructure of gypsum rocks, which influences their tensile strength more evidently after compression. Although the fiber striated structure of fiber gypsum is relatively compact, it is easily separated under tensile stress. In addition, this arc-shaped striation structure distribution peeled and stretched along the interlayer, leading to an arc-shaped fracture line during rock fracture. Alabaster has large blocky characteristics that cause easy stress concentration and failure under loading conditions, which contributes to the lowest tensile strength. The stress propagation path in the rock samples usually traverses the direction of the least work. Therefore, as the fracture line extends, large particles are bypassed, causing the zigzag fracture line and uneven particle size distribution of the damaged particles. Rock samples with less destroyed vein skeleton retained a good integrity. Transparent gypsum has a relatively uniform particle size distribution after failure, proving the more sufficient and higher energy release; thus, tensile strength was relatively high. Therefore, based on the microstructure analysis of the gypsum rocks, the strength of ordinary gypsum, transparent gypsum, fiber gypsum, and alabaster decreased during fracture, which is consistent with the experimental results.

Rock failure is the accumulation and release of internal energy. The work done by an external force causes the complete breakage of the rock mass into smaller particles. As the space between the particles expand after energy release, the external force exerts more work, thereby internal pores develop at a higher degree^50–52^. In addition, there is more energy stored in the rock, more obvious micro failure characteristics, and larger tensile strength. This is evident in the increased number of micro particles of alabaster, transparent gypsum, and ordinary gypsum in the same statistical area and the full development of pore space, confirming the increased tensile strength of gypsum rocks.

## Conclusions

In this paper, the content and microstructure of four kinds of gypsum rock—transparent gypsum, ordinary gypsum, fiber gypsum, and alabaster—were analyzed by XRF, XRD, SEM, and Brazilian split experiments. The influence of the composition and microstructure on the tensile strength and failure characteristics of the gypsum rocks and their mechanisms were investigated. The specific conclusions are as follows.

The main components of alabaster, fiber gypsum, transparent gypsum, and ordinary gypsum is found to be calcium sulfate dihydrate (CaSO_4_·2H_2_O) with a calculated content 72.78%, 72.72%, 72.57%, and 71.51%, respectively, and the grain size decreasing accordingly. For transparent gypsum rocks, the higher the CaSO_4_·2H_2_O content, the greater the peak value of its first main diffraction peak.

The tensile strength and density of alabaster, fiber gypsum, transparent gypsum, and ordinary gypsum decrease with the increase of CaSO_4_·2H_2_O content. In addition, there may be a negative correlation between the tensile strength and the content of CaSO_4_·2H_2_O, that is the higher the CaSO_4_·2H_2_O content, the lower the tensile strength of gypsum rock.

The four types of gypsum rocks have different fracture structural characteristics. In a macroscopic scale, fiber gypsum fracture line exhibits an arc shape that traverses the specimen while that of ordinary and transparent gypsums vertically traverses the center of the specimen. Alabaster has a zigzag line fracture with intergranular or trans-granular distribution. In contrast, in a microscopic scale, a stripe structure is observed for fiber gypsum, the pore development degree of alabaster, transparent gypsum, and ordinary gypsum gradually increases and the diameter of the destroyed particles decreases accordingly and uniformly.

Based on the molecular arrangement, the higher the CaSO_4_·2H_2_O content, the more evident the layered characteristics of the gypsum rock, and the larger the grain size, thereby the lower the tensile strength. The number of destroyed particles of alabaster, transparent gypsum, and ordinary gypsum is directly proportional to its tensile strength. Particularly, a larger tensile strength has a smaller particle diameter distribution interval.
